# An α5 GABAA Receptor Inverse Agonist, α5IA, Attenuates Amyloid Beta-Induced Neuronal Death in Mouse Hippocampal Cultures

**DOI:** 10.3390/ijms21093284

**Published:** 2020-05-06

**Authors:** Chitra Vinnakota, Karan Govindpani, Warren Perry Tate, Katie Peppercorn, Praju Vikas Anekal, Henry John Waldvogel, Richard Lewis Maxwell Faull, Andrea Kwakowsky

**Affiliations:** 1 Centre for Brain Research, Department of Anatomy and Medical Imaging, Faculty of Medical and Health, Sciences, University of Auckland, Auckland 1023, New Zealand; c.vinnakota@auckland.ac.nz (C.V.); k.govindpani@auckland.ac.nz (K.G.); h.waldvogel@auckland.ac.nz (H.J.W.); rlm.faull@auckland.ac.nz (R.L.M.F.); 2 Department of Biochemistry, University of Otago, Dunedin 9054, New Zealand; warren.tate@otago.ac.nz (W.P.T.); katie.peppercorn@otago.ac.nz (K.P.); 3 Biomedical Imaging Research Unit, Faculty of Medical and Health Sciences, University of Auckland, Auckland 1023, New Zealand; p.anekal@auckland.ac.nz

**Keywords:** Alzheimer’s disease, GABA, α5 GABAA receptors, α5IA

## Abstract

Alzheimer’s disease (AD) is a progressive neurodegenerative disorder for which no cognition-restoring therapies exist. Gamma-aminobutyric acid (GABA) is the primary inhibitory neurotransmitter in the brain. Increasing evidence suggests a remodeling of the GABAergic system in AD, which might represent an important therapeutic target. An inverse agonist of α5 subunit-containing GABAA receptors (α5GABAARs), 3-(5-Methylisoxazol-3-yl)-6-[(1-methyl-1,2,3-triazol-4-yl)methyloxy]-1,2,4-triazolo[3–*a*]phthalazine (α5IA) has cognition-enhancing properties. This study aimed to characterize the effects of α5IA on amyloid beta (Aβ_1–42_)-induced molecular and cellular changes. Mouse primary hippocampal cultures were exposed to either Aβ_1-42_ alone, or α5IA alone, α5IA with Aβ_1–42_ or vehicle alone, and changes in cell viability and mRNA expression of several GABAergic signaling components were assessed. Treatment with 100 nM of α5IA reduced Aβ_1–42_-induced cell loss by 23.8% (*p* < 0.0001) after 6 h and by 17.3% after 5 days of treatment (*p* < 0.0001). Furthermore, we observed an Aβ_1-42_-induced increase in ambient GABA levels, as well as upregulated mRNA expression of the GABAAR α2,α5,β2/3 subunits and the GABABR R1 and R2 subunits. Such changes in GABARs expression could potentially disrupt inhibitory neurotransmission and normal network activity. Treatment with α5IA restored Aβ_1-42_-induced changes in the expression of α5GABAARs. In summary, this compound might hold neuroprotective potential and represent a new therapeutic avenue for AD.

## 1. Introduction

Alzheimer’s disease (AD) is a chronic neurodegenerative disorder associated with a progressive loss of neuronal and synaptic density, which clinically manifests as a gradual decline in memory and cognitive function. Aside from amyloid-β (Aβ) plaques and neurofibrillary tangles, which are the characteristic neuropathological hallmarks of AD, the dysfunction of several neurotransmitter systems, including the inhibitory γ-aminobutyric acid (GABA) system, has been associated with the development and progression of AD [1,2,3,4,5,6]. Dysfunction in the glutamatergic and cholinergic systems, which contribute to the excitatory aspect of the excitatory/inhibitory (E/I) balance, has long been implicated in AD pathogenesis. The significant loss of cells in these systems and the disruption of their molecular components results in a disturbed E/I balance in the AD brain, which could underlie the cognitive deficits that are characteristic of the condition [7]. At present, all five drugs approved by the US Food and Drug Administration for the symptomatic treatment of AD are targeted towards these systems—including the acetylcholinesterase inhibitors donepezil, rivastigmine and galantamine, and the N-methyl-D-aspartate (NMDA) receptor antagonist memantine [8,9,10]. These drugs, however, provide marginal clinical benefit only and do not address the underlying causes of the disease. Therefore, there is an urgent need for the identification of novel therapeutic targets. Several studies have already shown that neurotoxicity induced by Aβ is mediated via an excitotoxic pathway involving accumulation of glutamate and over-activation of the NMDA receptor, making neurons more vulnerable to damage and death [11,12,13]. It has been hypothesized that targeting the GABAergic system could reduce neuronal vulnerability to excitotoxic damage by restoring the E/I balance [14,15]. GABAA receptor (GABAAR) positive allosteric modulators, which are compounds that enhance GABA action might protect against excitotoxicity occurring in AD through their anti-glutamatergic action [10,16]. On the other hand, GABAAR negative allosteric modulators act by decreasing the effect of GABA at its receptor, decreasing the excessive tonic conductance caused by the potentially elevated extrasynaptic GABA levels in the AD brain [10,17].

Studies have shown an age-dependent decrease in GABA currents in AD, along with changes in protein and mRNA levels of various GABAR subunits and GABA transporters [2,4,5,18]. These are most likely not just compensatory alterations, but reflect the reorganization of neuronal circuits and are critical to produce a stable neuronal network [6]. Given the role of GABA as the main inhibitory neurotransmitter, and its involvement in maintaining the homeostatic balance between E/I signaling in the central nervous system, any pathological change in this system might contribute to AD pathogenesis, neurodegeneration, and subsequent cognitive decline seen in patients. It has been hypothesized that disruption to GABAergic signaling might even be the trigger in a cascade of events ultimately culminating in the widespread dysfunction of neuronal networks and death of neurons seen in AD [19,20]. The GABAergic hypothesis of AD posits that aberrant GABAergic transmission leads to uncontrolled firing in the many neuronal populations innervated by inhibitory neurons, resulting in impairments in communication between neuronal networks involved in learning and memory, ultimately resulting in the characteristic cognitive impairments associated with AD [19]. Besides acting on synaptic receptors, GABA also mediates its effects through extrasynaptic receptors and activation of these receptors by increases in ambient GABA levels may also be involved in network dysfunction [17,21,22]. In the hippocampus, ambient GABA levels activate extrasynaptic α5 subunit-containing GABAA receptors (α5GABAARs), which are thought to mediate tonic inhibition [23,24]. An increase in ambient GABA levels leads to persistent and continuous activation of these receptors and generates a chronic hyperpolarizing conductance, ultimately affecting the excitability of neurons in the hippocampus, thus contributing to the alteration of the E/I balance and cognitive dysfunction in AD [4]. In a previous study, we have reported that Aβ induced an increase in tonic GABAergic current in the CA1 pyramidal neurons, along with significant neuronal loss and cognitive decline [21].

Previous studies have shown that α5GABAARs have potential as therapeutic targets to allay cognitive impairment and neuropsychiatric symptoms [25,26,27]. The last two decades have seen the development of several compounds, both positive and negative allosteric modulators, which target and modulate the function of the α5GABAARs, and show promise in improving cognition in neurological conditions in both animal and human studies [27,28,29,30]. However, further research is required to fully understand the mechanisms of action of these drugs and test their therapeutic potential, specifically for AD. This, in combination with the finding that α5GABAARs are relatively preserved in the AD hippocampus, led us to suggest for the first time that these receptors might be an important drug target in AD [2,31]. α5GABAARs are thought to be the key GABAAR subtype involved in modulating learning and memory due to their abundance in the hippocampus, where they comprise approximately a quarter of the GABAAR population [32,33,34]. Furthermore, it was found that α5 subunit knockout mice perform significantly better in spatial memory tasks, whilst mice with selective reduction of the α5 subunit in hippocampal pyramidal cells showed improvements in associative learning [23,35]. On the other hand, when there is an upregulation of α5GABAARs, such as in the dentate gyrus of 5xFAD mice, enhanced tonic inhibition and impairments in spatial memory are observed [17]. The precise neural mechanisms that underlie improvements in cognition associated with decreased α5GABAAR function have yet to be elucidated. However, these results suggest that selective targeting of α5GABAARs with a negative modulator could provide therapeutic benefit in AD by increasing cognitive function.

Previous studies conducted in rodents and healthy human subjects have indeed shown that inverse agonists of α5GABAARs display nootropic properties [29,36]. Alpha5IA {3-(5-methylisoxazol-3-yl)-6-[(1-methyl-1,2,3-triazol-4yl)methyloxy]-1,2,4-triazolo [3,4-a]phthalazine} is an α5GABAAR-selective inverse agonist with well-characterized in vitro and in vivo pharmacological properties [30,37]. The compound was shown to significantly enhance long-term potentiation and cognition in wild-type mice without inducing convulsions, seizures or anxiety, or altering motor function [30,38]. Furthermore, in a genetic mouse model of Down syndrome, α5IA was found to improve learning and memory outcomes [39]. When tested in humans, α5IA attenuated ethanol-induced impairment of word recall in healthy, young volunteers, with the greatest effect observed in subjects who were most impaired by ethanol [36]. This provides further support for the key role of the α5GABAAR in memory function and also implies that α5IA may be able to enhance cognition under certain conditions [37].

Inverse agonists selective for α5GABAARs, such as α5IA, therefore appear to be promising cognition-enhancing drugs, but they are yet to be tested in in vitro or animal models of AD. This study examined the effects of α5IA in an *in vitro* mouse model of AD and found that it prevented Aβ_1-42_-induced cell loss. These findings and the promising pharmacological properties of such compounds warrant further research.

## 2. Results

### 2.1. Effect of α5IA on Aβ_1-42_-induced Cell Viability in Mouse Hippocampal Cultures

**Figure 1:**
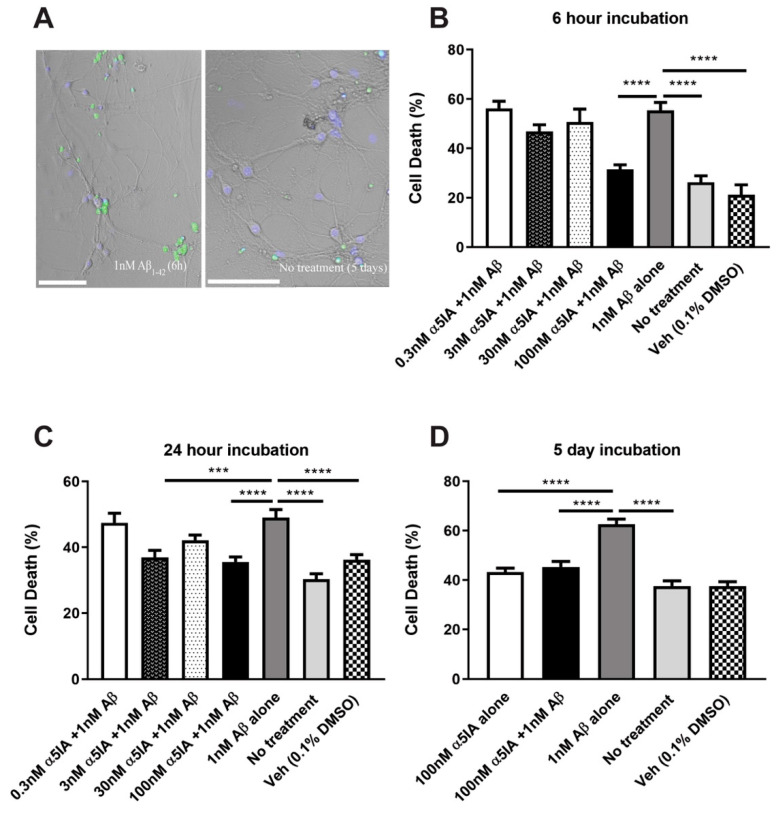
Cell death in mouse primary hippocampal cultures following treatment with 1 nM Aβ_1–42_ and 0.3 nM, 3 nM, 30 nM, and 100 nM of α5IA. (**A**) At 14 DIV, mouse primary hippocampal cells were stained with the ReadyProbes Live/Dead assay after 6 h treatment with 1 nM Aβ_1-42_ and without treatment for 5 days. Live nuclei (blue) and dead nuclei (green). Scale bars = 100 μM. (**B–D**) Quantification of the ReadyProbes Live/Dead assay showing percentage of cell death following treatment with various concentrations of α5IA for 6 h (**B**) and for 24 h (**C**). (**D**) Cell death following 5-day treatment with 100 nM α5IA and 1 nM Aβ_1-42_. Data are expressed as mean ± SEM. ***p < 0.001 ****p < 0.0001, One-way ANOVA with Bonferroni’s post hoc test, (*n* = 5–9).

Hippocampal cultures were treated with 0.3 nM, 3 nM, 30 nM, and 100 nM of the drug, α5IA, to investigate whether it would have any impact on Aβ_1–42_-induced cell death using the ReadyProbes Live/Dead assay. At the highest concentration, α5IA (100 nM) reduced Aβ_1-42_-induced cell death by 24% over a 6 h treatment (Figure 1B; *p* < 0.0001, *n* = 5). Treatment with lower concentrations of the drug for 6 h were not effective at increasing cell viability. To study the long-term effects of drug treatment, cell viability following treatment with 1 nM Aβ_1-42_ and 0.3, 3, 30 or 100 nM of α5IA for 24 was also measured. As with the short-term treatment, at a concentration of 100 nM, α5IA significantly reduced Aβ_1-42_-induced cell death, by 13% (Figure 1C; *p* < 0.0001, n = 6). The drug, α5IA, at 3 nM also ameliorated Aβ_1-42_-induced cell death by 12% after the 24 h treatment (Figure 1C; *p* = 0.0009, *n* = 5), although 30 nM (and 0.3 nM α5IA) had no effect. Cell viability after five days of treatment with 100 nM α5IA was also measured and revealed a decrease in Aβ_1-42_-induced cell death by 17% (Figure 1A,D; *p* < 0.0001, *n* = 9).

**Figure 2:**
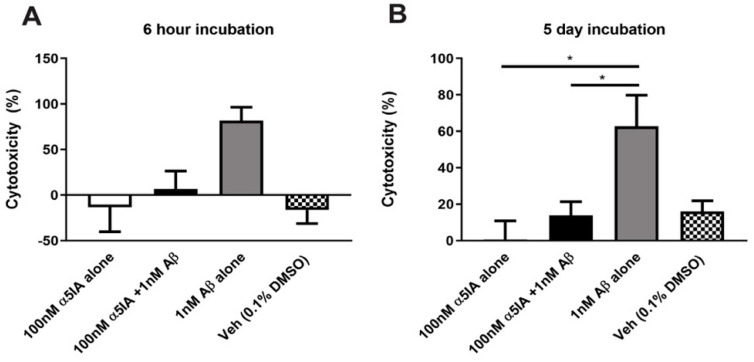
Cytotoxicity (%), measured by LDH release, in mouse primary hippocampal cultures following treatment with 100 nM α5IA and 1 nM Aβ_1-42_ for 6 h (**A**) and 5 days (**B**). Cells lysed with 1% Triton X-100 in maintenance media were used as the positive control. Values were expressed as a percentage of the positive control and normalized to untreated controls. Data is expressed as mean ± SEM. *p < 0.05, One-way ANOVA with Bonferroni’s post hoc test, (*n* = 5–8).

The lactate dehydrogenase (LDH) assay was used to measure cytotoxicity. After five days of treatment, cultures treated with 100 nM α5IA alone and cultures treated with both 100 nM α5IA and 1 nM Aβ_1–42_ had decreased cytotoxicity compared to cultures treated with 1 nM Aβ_1-42_ alone (Figure 2B; 100 nM α5IA alone vs. 1 nM Aβ_1-42_ alone p = 0.01; 100 nM α5IA and 1 nM Aβ_1-42_ vs. 1 nM Aβ_1-42_ alone *p* = 0.03, *n* = 5–8). There was no significant change in cytotoxicity following treatment with 100 nM α5IA for 6 h (Figure 2A; *n* = 5–8).

**Figure 3:**
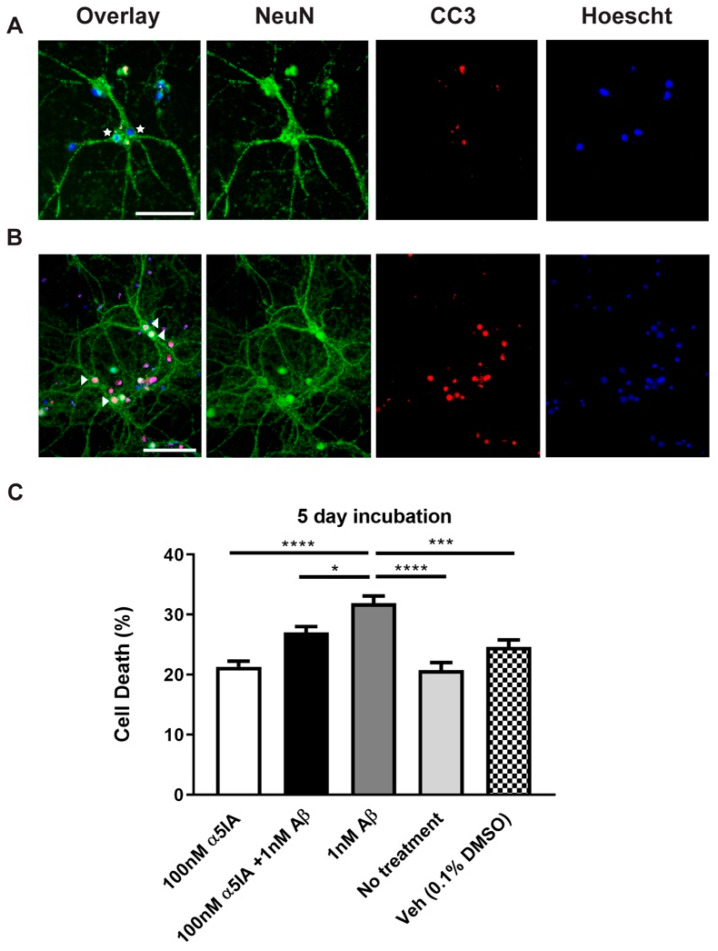
Apoptotic cell death in mouse primary hippocampal cultures following treatment with Aβ_1-42_ and α5IA. (**A/B**) Photomicrographs of mouse primary cultures stained with neuronal marker, NeuN (green) and apoptotic marker cleaved caspase-3 (CC3; red) after treatment with 100 nM α5IA + 1 nM Aβ_1–42_ (A) and 1 nM Aβ_1–42_ (B) for 5 days. Nuclei are counterstained with Hoechst 33342. Live neuronal nuclei (blue; star) and dead neuronal nuclei (pink/white; arrows). Scale bars = 100 μM. (**C**) Quantification of the CC3 assay showing percentage of cell death. The data is graphed as mean ± SEM. *p < 0.05, ***p < 0.001, ****p < 0.0001, One-way ANOVA with Bonferroni’s post hoc test, (*n* = 8–12).

To further evaluate cell viability, primary cultures were co-stained with NeuN and the apoptotic marker cleaved-caspase 3 (CC3), following treatment with Aβ_1-42_ alone, α5IA alone or Aβ_1–42_ with α5IA, to detect and quantify the number of apoptotic neuronal cells. Treatment with a combination of 100 nM α5IA and 1 nM Aβ_1-42_ resulted in a significant decrease in apoptotic cell death compared with Aβ_1-42_-treated cultures (Figure 3C; *p* = 0.01, *n* = 12). This indicates trends similar to those observed in the previous cell viability assays.

### 2.2. Aβ_1–42_ -induced Changes in GABA Levels in Mouse Hippocampal Cultures

**Figure 4:**
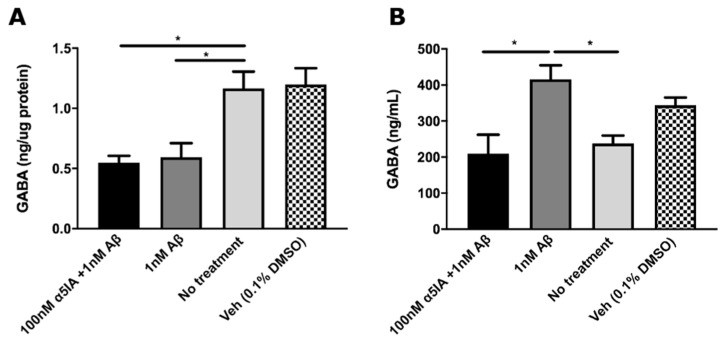
Intracellular (**A**) and extracellular (**B**) GABA concentrations in mouse primary hippocampal cultures 5 days after treatment with Aβ_1–42_ and α5IA + Aβ_1-42_ as measured by ELISA. Intracellular GABA levels were normalized to protein content measured using DC assay. Extracellular refers to GABA in the culture medium. Data is expressed as mean ± SEM. * *p* < 0.05, One-way ANOVA with Bonferroni’s post hoc test, *n* = 3 for each experimental group with triplicate measurements.

Once it was established that Aβ_1-42_ induced cell death, cultures were treated with Aβ_1-42_ for 5 days to study the effect on GABA levels. Following 5 days of treatment, cultures treated with 1 nM Aβ_1–42_ alone displayed a significant decrease in intracellular GABA concentration compared to untreated controls (Figure 4A; *p* = 0.04, *n* = 3). Changes in the levels of extracellular GABA released into the culture media were also measured. Treatment with 1 nM Aβ_1-42_ increased extracellular GABA levels compared to untreated controls (Figure 4B; *p* = 0.02, *n* = 3). In cultures treated with both Aβ_1–42_ and α5IA, there was no significant difference in the intracellular GABA concentration compared to cultures treated with Aβ_1-42_ alone (Figure 4A; *n* = 3). There was, however, a decrease in the concentration of extracellular GABA compared to Aβ_1-42_-treated cultures (Figure 4B; *p* = 0.02, *n* = 3).

### 2.3. Effect of α5IA on Aβ_1–42_ -induced Changes on the Expression of GABAergic Signaling Components in Mouse Hippocampal Cultures

**Figure 5:**
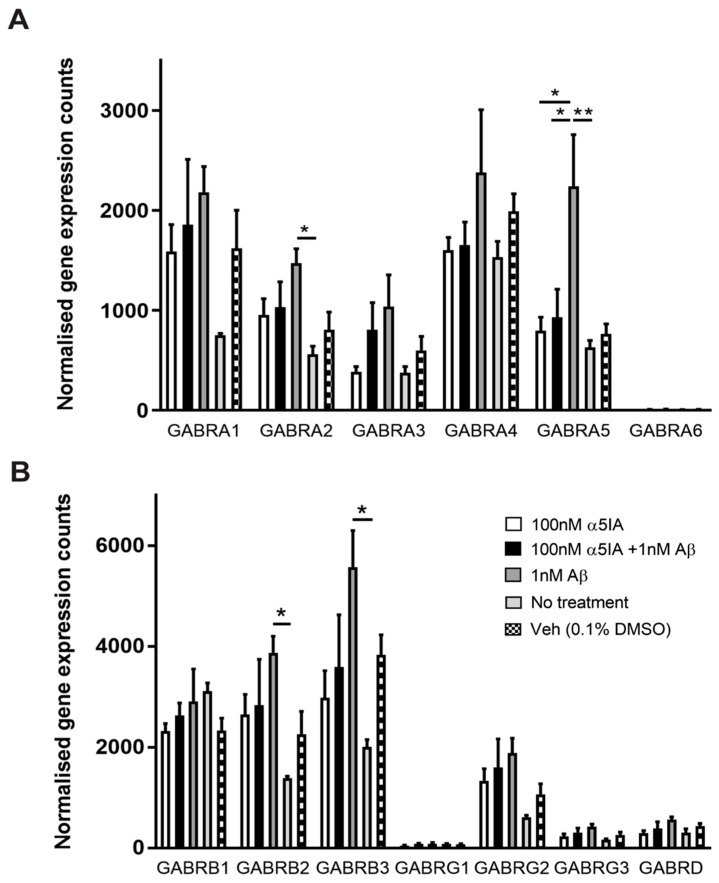
Expression of GABAergic signaling components in mouse primary hippocampal cultures 5 days after treatment with 100 nM α5IA and/or 1 nM Aβ_1–42_. A comprehensive analysis of changes in mRNA expression was performed using the NanoString nCounter gene expression assay. (**A**) Expression of GABAA receptor alpha subunits. (**B**) Expression of GABAA receptor beta, gamma, and delta subunits. Values are normalized to six housekeeping genes. Data is expressed as mean ± SEM. **p < 0.01, *p < 0.05, compared to negative controls, One-way ANOVA with Bonferroni’s post hoc test for each gene. *n* = 4–5 for each experimental group.

**Figure 6:**
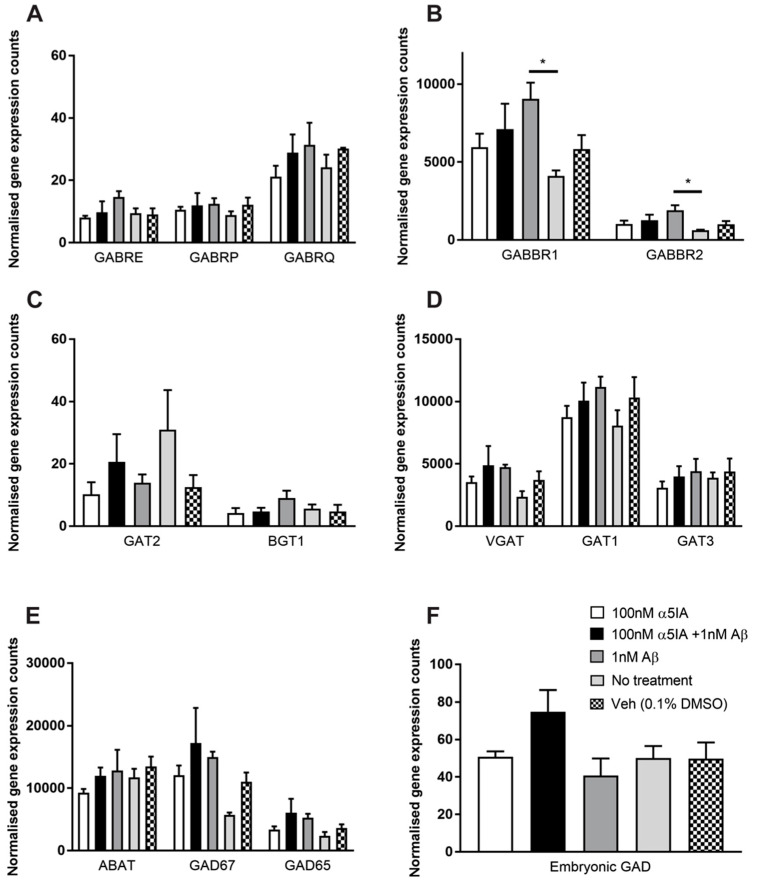
Expression of GABAergic signaling components in mouse primary hippocampal cultures 5 days after treatment with 100 nM α5IA and/or 1 nM Aβ_1–42_. A comprehensive analysis of changes in mRNA expression was performed using the NanoString nCounter gene expression assay. (**A**) Expression of GABAAR epsilon, pi, and theta subunits. (**B**) Expression of GABAB receptor 1 and GABAB receptor 2 subunits. (**C**) Expression of GABA transporters (GAT) GAT2 and betaine transporter 1 (BGT1). (**D**) Expression of vesicular GABA transporter (VGAT) and GAT1 and GAT3. (**E**) Expression of GABA catabolizing enzyme; GABA transaminase (ABAT) and GABA synthesizing enzymes; glutamic decarboxylase (GAD65 and GAD67). (**F**) Expression of GABA synthesizing enzyme embryonic GAD. Values are normalized to 6 housekeeping genes. Data is expressed as mean ± SEM. *p < 0.05, compared to negative controls, One-way ANOVA with Bonferroni’s post hoc test for each gene. *n* = 4-5 for each experimental group.

The expression levels of components of the GABA signaling system were studied in mouse hippocampal neuronal cultures, to investigate the effects of treatment with Aβ_1–42_ and α5IA (Table 1). Following treatment with 1 nM Aβ_1–42_, RNA expression of the GABAAR α2 subunit was significantly increased compared with untreated controls (Figure 5A; *p* = 0.02, *n* = 5). Notably, the α5 subunit was also significantly upregulated (Figure 5A; *p* = 0.007, *n* = 5). There was a significant reduction in the expression of the α5 subunit following treatment with 100 nM α5IA alone compared with Aβ_1–42_ -treated cells (Figure 5A; *p* = 0.04, *n* = 5). Cells treated with both α5IA and Aβ_1–42_ also showed decreased expression of the α5 subunit compared with cells treated with Aβ_1–42_ alone (Figure 5A; *p* = 0.05, *n* = 5). RNA expression of the GABAAR β2 subunit was also increased in Aβ_1–42_-treated cells compared with untreated control (Figure 5B, *p* = 0.05, *n* = 5), and this was also the case for the β3 subunit (Figure 5B, *p* = 0.02, *n* = 5). No significant changes in expression levels were observed for other GABAAR subunits following treatment with Aβ_1–42_ and/or α5IA, although the α1 and γ2 subunits showed a trend towards increased RNA expression in Aβ_1–42_-treated cultures compared with untreated cultures (Figure 5A-B, Figure 6A; *n* = 5).

The expression of the GABAB receptor 1 subunit (GABABR1) increased significantly after treatment with 1 nM Aβ_1–42_ compared with untreated controls (Figure 6B; *p* = 0.05, *n* = 5). Treatment with 1 nM Aβ_1–42_ also increased the expression levels of GABAB receptor 2 subunit (GABABR2) compared with untreated controls, and this was unaffected by treatment with α5IA (Figure 6B; *p* = 0.03, *n* = 5).

The expression levels of the GABA transporters (BGT1, GAT1-3 and VGAT), catabolizing enzymes (GABA transaminase; ABAT) and GABA synthesizing enzymes (glutamic acid decarboxylase; GAD65, GAD67 and embryonic GAD) were not significantly altered following Aβ_1–42_ or α5IA treatment (Figure 6C–F; *n* = 5). Although, of note is the trend towards increased VGAT expression in Aβ_1–42_-treated cultures compared to controls, which may relate to the increase in extracellular GABA seen in Figure 4B (Figure 6D; ns, *n* = 5).

## 3. Discussion

The present study is the first to examine the effects of α5IA, an α5GABAA receptor inverse agonist, in an *in vitro* mouse model of AD. This study uncovered the novel finding that α5IA is potentially neuroprotective. Aβ_1–42_-induced cell death was significantly reduced in primary mouse hippocampal cultures following treatment with α5IA. This effect occurred as early as 6 h after drug treatment and was also present after 5 days of treatment.

We found that levels of GABA were altered following Aβ_1–42_ treatment, with an increase in released GABA and a decrease in intracellular GABA in primary hippocampal neuronal cultures, suggesting that increased GABA secretion might lead to lower intracellular concentration. The Aβ_1–42_-induced increase in ambient GABA could result in increased binding and activation of α5GABAARs, leading to increased tonic inhibition, ultimately disrupting the E/I balance and causing cell death. In a previous study, we have indeed demonstrated that tonic GABAergic current is increased in the CA1 pyramidal neurons of Aβ_1–42_-injected mice, which potentially contributes to the observed neuronal loss and cognitive decline [21,40]. Other studies also reported that neurotransmitters such as GABA play an important role in regulating neuronal survival and apoptosis [41,42,43]. A study using a rat model of post-traumatic stress disorder found that a disequilibrium between glutamate and GABA promoted the apoptosis of hippocampal neurons [44]. Thus, it is possible that the aberrant change in GABA levels following treatment with Aβ_1–42_ plays a role in the observed increase in cell death. Further, it is possible that α5IA, which binds to α5GABAARs and acts as an inverse agonist, works to restore the E/I balance, thus preventing cell death. We also found that α5IA appeared to decrease the Aβ_1–42_-induced increase in ambient GABA levels. In human AD patients, multiple studies have reported reductions in GABA levels in the hippocampus [45,46], although such results are contentious and sometimes inconsistent [1]. This is similar to the trend we observed for intracellular GABA levels in our *in vitro* model. Due to technical challenges, alterations in extracellular GABA levels, however, have not yet been measured in human AD brains. *APP/PS1* mice which display significant deficits in synaptic plasticity and learning and memory were found to display significantly elevated levels of GABA compared with wild-type mice [22]. The study concluded that anomalous alterations to GABAergic neurotransmission lead to a chronic E/I imbalance, which underpins the synaptic dysfunction in AD [22]. In *APP/PS1* mice, there was a decrease in the GABAergic inhibition of hyperactive neurons and an increase in the inhibition of hypoactive neurons in the CA1 region of the hippocampus [1,47]. This led to hyperexcitability which occurred prior to plaque formation, implying that an altered E/I balance might play a key role in the pathogenesis of AD [1,47]. Restoration of this balance by reducing neuronal hyperexcitability was found to rescue neuronal dysfunction and resulted in behavioral improvements in the mice [48].

We also hypothesized that the changes in cell viability in our *in vitro* AD model following treatment with α5IA may be linked to alterations in the expression of components of the GABAergic signaling system, as previous studies have indicated that there are complex AD-related changes in the expression of several GABAergic components [1,2,49,50]. We observed Aβ-induced changes in the expression of the α2, α5, β2, and β3 GABAAR subunits and both the R1 and R2 subunits of the GABABR. The subunit composition of the GABAAR determines the receptor’s functional activity and binding affinity for GABA. Certain subunit compositions result in altered GABA binding affinity and receptor function, determining the unique roles of these GABAAR assemblies in inhibitory signaling [51].

We observed an Aβ_1–42_-induced increase in the expression of the α5 subunit in our *in vitro* AD model. It has previously been shown that the expression of α5GABAARs is dynamic and may be altered in mouse models of AD, leading to deteriorating memory performance [17,52]. It has also been shown that α5GABAAR expression is altered in the inflammatory state in mice, with additional implications for memory performance [52]. Moreover, recent studies have shown that the α5 subunit is upregulated in the CA1 region of the human AD hippocampus [2]. As the α5 subunit is present in approximately 25% of hippocampal GABAARs, an alteration in the expression of this subunit would have wide-ranging implications for receptor composition and function in this region. The extra-synaptic high affinity α5GABAARs can be persistently activated by low concentrations of ambient GABA to generate tonic inhibition, which is thought to be important for learning and memory [53,54]. Therefore, any alterations in this receptor’s expression might play a role in the cognitive decline seen in AD. As mentioned earlier, previous studies directly linked the anomalous over-expression of the α5 subunit with cognitive dysfunction in mice [17]. Conversely, the absence or a decrease in the expression or activity of α5GABAARs has been found to enhance cognition [23,35]. Thus, it has been hypothesized that the upregulation of this receptor subtype in AD could contribute to some of the cognitive symptomatology of the disease. Treatment with α5IA was shown to restore hippocampal-dependent learning and memory function in various animal models as well as in healthy humans [30,36]. However, the mechanism behind the memory-enhancing effects are unknown. In this study, we have shown that the restorative effects of α5IA might involve the reversal of Aβ-induced α5 subunit upregulation. The upregulation of the α5 subunit in AD may be a compensatory mechanism in response to altered GABAergic tone and may act to enhance tonic inhibition.

The α5 subunit is most commonly co-expressed with the β3 and γ2 subunits [55,56]. We found that the increased expression of the α5 subunit following application of Aβ correlated with significantly increased expression of the β3 subunit. We also found a significant upregulation of the α2 and β2 subunits in Aβ-treated cultures compared with controls. The remaining subunits stayed largely unaltered in expression and appeared to be well preserved in our *in vitro* AD model. Treatment with α5IA reversed the Aβ-induced increase of the α5GABAAR subunit expression and the β3 subunit showed a similar non-significant trend towards decreased expression. These findings suggest an altered Aβ-induced expression of the α5 subunit containing GABAARs that might be ameliorated by α5IA treatment.

GABABRs are heterodimeric metabotropic receptors, comprising two subunits termed R1 and R2. In our study, we found an upregulation of both the R1 and R2 subunits of the GABABR in mouse hippocampal cells treated with Aβ compared with untreated controls. The GABABRs are also reported to be highly sensitive to tonic GABA currents and when activated post-synaptically have been shown to act via a pathway involving G proteins, adenylate cyclase, and cAMP-dependent protein kinases to enhance tonic GABA currents through extrasynaptic GABAARs, including the α5GABAARs [57,58,59]. Treatment with α5IA did not have a significant effect on Aβ-induced GABABR upregulation.

Although differences in mRNA expression of other components of the GABAergic system were examined between treatment groups, no significant differences were identified for either the GABA synthesizing enzymes or the GABA transporters. This precludes the involvement of GAD expression changes on the altered intracellular and extracellular GABA levels in response to Aβ_1–42_.

## 4. Materials and Methods

### 4.1. Animals

All procedures were approved by the University of Auckland Animal Ethics Committee. Mice were housed at the University of Auckland Vernon Jansen Unit under standard laboratory conditions. Early postnatal (P0) male C57/BL/6 mice were used to establish hippocampal primary cultures.

### 4.2. Hippocampal Primary Cell Culture

All culture procedures were performed according to the protocol established by Beaudoin and colleagues with slight modifications [60]. Briefly, P0 C57/BL/6 male mouse pups were euthanized by decapitation, the hippocampus was dissected, and any surrounding meninges and blood vessels were removed. Hippocampi were washed in Hank’s balanced salt solution (HBSS; Invitrogen 14175095, New York, NY, USA) supplemented with 1 mM sodium pyruvate, 10 mM HEPES buffer and 0.1% glucose, and then enzymatically dissociated using 0.25% trypsin (Invitrogen) in Basal Medium Eagle (Invitrogen, 21010046, New York, NY, USA). Hippocampi were then mechanically dissociated using a fine glass pipette to obtain a homogeneous cell suspension. Cells were plated into poly-D-lysine (Sigma, P6407, Saint Louis, MO, USA) coated culture dishes and incubated at 37 °C in a 5% CO_2_/95% O_2_ cell culture incubator for 5 h to allow cells to adhere to the plastic culture surface. After this, media from the culture dish containing unattached cells and debris was removed and replaced with Neurobasal medium (Invitrogen, 21103049, New York, NY, USA) supplemented with B27 supplement (Invitrogen, 17504044, New York, NY, USA), 2 μg/mL gentamicin (Invitrogen, 15140122) and 2 mM glutamine (Invitrogen, 25030081, Paisley, Scotland). Half the medium was exchanged every 4 days. Neurons could be maintained up to 4 weeks, during which time they developed well differentiated axons and dendrites and formed functional synaptic connections. 5 μM of cytosine arabinofuranoside (araC; Sigma, 251010, Darmstadt, Germany) was added to the culture medium for 24 h at 3 days in vitro (DIV) to inhibit the proliferation of glia and minimize the number of astrocytes in the culture.

### 4.3. Aβ_1–42_ Preparation

Aβ_1–42_ was produced as a recombinant protein fused to maltose-binding protein (MBP) with a proteolytic cleavage site for Factor X protease between the two segments (Wilson C. MSc Thesis, University of Otago, 2007) [21,40,61]. The soluble recombinant fusion protein was then expressed at high concentrations in *Escherichia coli* and purified on an amylose column to which the MBP segment of the protein binds. After binding to amylose resin, the pure fusion protein was eluted from the resin with maltose and concentrated by ammonium sulfate precipitation. The carrier MBP was cleaved off the fusion protein by Factor X protease, and the released Aβ_1–42_ isolated and further purified by hydrophobic chromatography with 0–50% *v/v* acetonitrile/0.1% *v/v* TFA, using FPLC. The fractions containing pure Aβ_1–42_ were detected immunologically with an antibody against residues 17–24 of Aβ_1–42_ and lyophilized to remove solvent. Mass spectrometry was used to confirm the expected molecular ion for the desired product. The concentration of the protein fragment was determined with the bicinchoninic acid assay at 60 °C for 30 min. Before adding Aβ_1–42_ to the culture medium, it was ‘aged’ by incubating at 37 °C for 48 h to facilitate the formation of toxic soluble aggregates of misfolded Aβ_1-42_, which was confirmed by SDS/PAGE and by non-dissociating PAGE [21,40].

### 4.4. Drug Treatments

At 14 DIV, when the cultured murine hippocampal neurons represent an ‘adult’ state of development, 1 nM of aged neurotoxic Aβ_1–42_ oligomers (dissolved in MilliQ water) was added to the culture medium for various lengths of time (6 h, 24 h or 5 days) to create an in vitro mouse model of AD. The inverse agonist α5IA (AdooQ Bioscience, A14343, Irvine, CA, USA) was diluted in 10% DMSO and neurobasal medium to make a 10 μM stock solution. The stock solution was heated to 60 °C and sonicated for 10 - 15 min. Cell viability following treatment with 1 nM Aβ_1–42_ and 0.3 nM, 3 nM, 30 nM or 100 nM α5IA for 6 h, 24 h, and 5 days was assessed.

### 4.5. Measuring Cell Viability Using the ReadyProbes Cell Viability Imaging Kit

Cell viability at 6 h, 24 h, and 5 days post-treatment with Aβ1–42 and/or α5IA was determined using the ReadyProbes Cell Viability Kit (Thermo Fisher, R37609, Eugene, OR, USA), as per the manufacturer’s instructions. Wells were randomly assigned as controls or for treatment with different concentrations of the drug (0.3 nM, 3 nM, 30 nM, and 100 nM) and 1 nM Aβ_1–42_ or with 1 nM Aβ_1–42_ alone for 6 h, 24 h, or 5 days. Controls included neurons treated with the vehicle (0.1% DMSO) and untreated control neurons, which were exposed to the same volume of culture medium. At least 5 wells were allocated per experimental group. One drop each of the NucBlue^®^ Live reagent and NucGreen^®^ Dead reagent were added to 500 μL of the culture medium in each well, in order to label the cells. Cell culture plates were then returned to the incubator for 15 min. The NucBlue^®^ Live reagent stains the nuclei of all cells (live and dead) while the NucGreen^®^ Dead reagent stains only the nuclei of dead cells. Cells were imaged on the EVOS™ microscope (Invitrogen, Carlsbad, CA, USA) using the 360/460 nm and 504/523 nm filter sets. Five images were taken at random sites across each well at a magnification of 20×, covering equal areas across all treatment groups. The total cell count, as determined by nuclei fluorescing blue, versus the number of dead cells, whose nuclei fluoresce green, was determined manually, counting only those cells with a neuronal morphology.

### 4.6. Measuring Cell Viability Using Cleaved-caspase 3 (CC3) Assay

Co-immunostaining for CC3, an apoptotic marker, and NeuN, a neuronal marker, was carried out to corroborate the key findings from the ReadyProbes assay using automated analysis to reduce the bias associated with manual counting. At least 8 wells were allocated to each experimental and control group. Neurons plated directly onto the plastic bottoms of 24-well culture plates were fixed at the appropriate time-points in 4% paraformaldehyde (PFA) solution, after which the fixed cells were washed in phosphate-buffered saline (PBS) and stored in 0.1% PBS-azide. Cells were washed in PBS containing 0.05% Tween 20 (PBT) and incubated in PBS containing 0.2% Triton X-100 (PBST) for 5 min at RT. The cells were again washed in PBT and then incubated overnight at 4°C in a solution containing 1% bovine serum albumin in PBT and primary antibodies (1:500 mouse NeuN, Millipore, MAB377, Burlington, NJ, USA; 1:500 chicken NeuN, Abcam, AB134014, Cambridge, MA, USA; 1:1000 rabbit CC3, Life Technologies, PA5-23921, Carlsbad, CA, USA). The following day, cells were washed in PBS and treated with Alexa Fluor secondary antibodies (1:500 goat anti-mouse 488, Invitrogen, A11001, Carlsbad, CA, USA; 1:500 goat anti-chicken 488, Abcam, AB150173, Cambridge, MA, USA; 1:500 goat anti-rabbit 594, Invitrogen, A11012; Carlsbad, CA, USA) for 1 h at room temperature (RT). Cells were incubated in 1:10,000 Hoechst 33,342 (Molecular Probes, H3570; diluted in PBT) for 5 min and imaged.

The ImageXpress® Micro XLS microscope (Version 5.3.0.1, Molecular Devices, San Jose, CA, USA) was used for image acquisition of the cultured cells. Images were taken at 16 well-separated sites across each well at 20× magnification (0.45 N.A., Plan Fluorite). Automated analysis was performed for quantification of stained cells using Image J software (Version 1.46r, National Institutes of Health). Briefly, images were color-combined using the MetaXpress® software (Version 5.3.0.1, Molecular Devices) to create a 16-bit composite image which was then imported into ImageJ and split into three channels. NeuN images were processed using 2 iterations of the TopHat filter (1: min [50] connectivity 8, 2: White top hat Disk Radius 5) from the MorphoLab plugin to clearly demarcate the bright NeuN puncta in neurons. The Otsu auto threshold was applied to segment these NeuN puncta and a closing function (MorphoJ: disk element radius 3) used to join these puncta into the cell body, producing the final neuronal mask. For Hoechst, after background subtraction (correcting for uneven illuminated background), the Otsu auto threshold was applied, and the watershed function used to separate neighboring nuclei and generate the final nuclei mask. To process the CC3 signal, background subtraction was performed followed by the Otsu auto threshold to segment the apoptotic bodies. This was dilated (itern-3 pixels-2) to produce the final caspase mask, as the segmented puncta area was a little smaller than the actual CC3 puncta. A size filter (min 20pixels) was applied to both the NeuN and CC3 masks to exclude small puncta noise and an AND function was applied with the nuclei mask to identify all NeuN cell bodies which contained nuclei (all neurons) as well as a further AND function between all neurons and CC3 mask to identify apoptotic neurons. This ImageJ macro was used to identify the percentage of apoptotic neurons in the total population of neurons.

### 4.7. Measuring Cell Viability Using the Lactate Dehydrogenase (LDH) Assay

To determine cytotoxicity, LDH released from the cytosol of damaged cells into the supernatant, was measured as per the manufacturer’s instructions (Roche, 11644793001, Basel, Switzerland). Briefly, cells were treated with 1 nM Aβ_1–42_ and 100 nM α5IA over 6 h or 5 days. All samples and controls were run in triplicate. A positive control was also prepared by lysing cells using the detergent Triton X-100 (1%) in maintenance medium. To obtain the cell-free supernatant, the 24-well culture plate containing the cells was centrifuged at 250 rpm for 10 min at RT and 100 μL of the supernatant was transferred to a new optically clear 96-well flat bottom plate. 100 μL of the freshly prepared reaction mixture was then added and the plate incubated at RT for 30 min. The absorbance of the samples was measured at 492 nm on a FLUOStar Optima plate reader (BMG Labtech, Ortenberg, Germany). The absorbance of cell-free culture media (background control) was subtracted from all values and the absorbance normalized between untreated controls (low cytotoxicity) and the positive control (100% cytotoxicity). Cytotoxicity (%) = ((Experimental value - low control)/(High control – low control)) X100.

### 4.8. Measurement of GABA Levels Uusing the Enzyme Linked Immunosorbent Assay (ELISA)

#### 4.8.1. Sample collection for ELISA

Mouse hippocampal neurons were treated with either 1 nM Aβ_1–42_ alone, with 1 nM Aβ_1–42_ and 100 nM α5IA, 0.1% DMSO (vehicle control) or left untreated (negative control) at 14 DIV and incubated at 37 °C for 5 days. Three T75 flasks (Thermo Fisher, NUN156499, Waltham, MA, USA) of hippocampal neurons were allocated per experimental group. Following the 5-day treatment, cells were harvested from each flask using 0.05% Trypsin-EDTA (0.5mM EDTA, 5.5mM glucose, 0.25% trypsin at pH 7.35, 1xPBS), transferred to a 15-mL tube and centrifuged at 700 rpm for 5 min. The supernatant was discarded, cells resuspended in 1xPBS, and centrifuged again at 700 rpm for 5 min to wash out excess media. The PBS was decanted and the cell pellet snap-frozen on dry ice and stored at -80 °C. The culture media was also collected, with two replicates per experimental group for analysis of extracellular GABA levels.

#### 4.8.2. Cell Homogenization and the Determination of Protein Concentration Using the Detergent-Compatible (DC) Assay

Cell pellets were retrieved from the -80 °C freezer for homogenization and to prepare the lysate. Samples were homogenized in lysis buffer (50 mM Tris, 2 mM EDTA, 1 mM PMSF, 1% protease inhibitors, Sigma, P8340, St. Louis, MO, USA) before being sonicated in ice-cold water for 4 min. A syringe with a 20G needle was used to homogenize samples, after which they were incubated on ice for 1 h before being centrifuged at 10,000 rpm for 10 min. The samples and standards were run in triplicate of 10 μL each and 9 blanks containing 180 μL of lysis buffer each were also included on a 96-well plate (Nunc, 167008). To each sample (except blanks), Reagent A and S (Biorad DC Assay Kit, USA, 500-0115) were added, followed by the light-sensitive Reagent B (Biorad DC Assay Kit, USA, 500-0115). Plates were incubated in the dark on a horizontal plate shaker for 3 min, before being incubated for a further hour at RT. Absorbance was measured at 750 nm on the FLUOStar Optima plate reader (BMG Labtech, Ortenberg, Germany). A standard curve was generated using the BSA standard dilution series. Protein concentrations were calculated by extrapolation from the standard curve and averaged across the triplicates. All samples were then diluted to the concentration of the most dilute sample (360µg/mL) to standardize them before being used for the ELISA.

#### 4.8.3. Quantitative Analysis of GABA Levels with ELISA

GABA concentration in cell culture was determined using an ELISA immunoassay kit as per the manufacturer’s instructions (LDN, BA E-2500, Nordhorn, Germany). All standards, controls, reagents and plates used were those provided with the kit. All samples, standards, and controls were run in duplicates. In brief, 100 μL of each sample type (undiluted standards, control or samples) and 100 μL of the diluent were added to each well of the extraction plate. The plate was covered with adhesive foil and agitated on a shaker at 600 rpm for 15 min at RT. The wells were washed with Milli-Q water and incubated for 5 min at RT on a shaker at 600 rpm, after which 400 μL of elution buffer was added to each well. The extraction plate was then covered and incubated for a further 10 min on the shaker at RT. Next, 100 μL of each extract was transferred to a new 48-well plate and used for derivatization. 10 μL of 0.1 M NaOH and 50 μL of the equalizing reagent (freshly prepared before each assay) was added to the wells, and the plates incubated on a shaker for 1 min at 600 rpm. 10μL of the D-reagent was added into each well, and the plate incubated on a shaker for 2 h at RT. Next, 150 μL of Q-buffer was added into each well and the plate shaken at 600 rpm for 10 min at RT. From each well, 50 μL was transferred to the GABA microtiter strips (pre-coated with antigen) and then 50 μL of GABA antiserum was added, mixed, covered, and incubated overnight at 4 °C on a shaker. The following day, the plate was washed thrice with wash buffer, after which the enzyme conjugate was added to each well, the plate covered, and incubated for 30 min again at RT with shaking. After another 3 washes, the substrate was added, and the plate covered in foil and incubated on a shaker for 30 min at RT. Finally, the stop solution was added with shaking and the absorbance read at 450 nm, using the Synergy 2 microplate reader (BioTek, Winooski, VT, USA). For the culture media samples, the GABA concentration was determined by comparing absorbance with a standard curve generated with known GABA concentrations. For all cell lysate samples, GABA concentration (ng/mL) was extrapolated from this standard curve and then divided by the protein content (36 µg) of the sample, with data expressed as nanograms of GABA per microgram of protein (ng GABA/µg protein).

### 4.9. Sample Collection, RNA Extraction and Quality Control

Sample collection for RNA expression analysis was as described for the ELISA but additionally included mouse hippocampal neurons treated with 100 nM α5IA alone. Five T75 flasks were allocated per experimental group. A Trizol-based protocol was used to extract RNA from the cell samples. Briefly, 150 μL of Trizol reagent (Invitrogen, 15596-026, Carlsbad, CA, USA) was added to each cell pellet to lyse and homogenize cells. Homogenized samples were incubated for 5 min before adding 30 μL of chloroform, shaking vigorously for 15 s and then incubating for 15 min at RT. Samples were then centrifuged at 13,500 rpm for 15 min at 4 °C. The upper aqueous phase was carefully transferred to a clean tube on ice and the RNA precipitated by adding isopropanol and incubating at RT for 10 min. The samples were centrifuged at 12,000 rpm for 10 min at 4 °C. The supernatant was removed carefully, and the sample washed twice with 150 μL of ice-cold 70% ethanol. The tubes were briefly vortexed and then centrifuged at 7,500 rpm for 5 min at 4 °C. The supernatant was decanted, and the pellet allowed to dry briefly. Samples were eluted in 15 μL of RNAse-free water. The concentration of the extracted RNA and the 260/230 and the 260/280 ratios were measured using a Nanodrop spectrophotometer (Thermo Fisher, 117127, Wilmington, NC, USA). The 260/230 ratios ranged from 1.5 to 2.3 and the 260/280 ratios from 1.73 to 2.3 across the samples. RNA quality was assessed using the Agilent 2100 Bioanalyzer (Agilent Technologies, G2947CA, Waldbronn, Germany) as per the manufacturer’s protocol to determine RNA integrity (RIN) values for each of the samples. RIN values ranged from 6.1 to 8.9 across the samples.

### 4.10. NanoString nCounter Analysis of RNA Expression

In total, 27 genes encoding various components of the GABAergic system were selected for RNA expression analysis using the nCounter gene expression analysis system (Table 2). Six housekeeping genes were also included, for normalization purposes, based on NanoString’s mouse neuropathology panels. The selected list of genes and the mRNA transcript accession numbers (NCBI Reference Sequence database) were sent to NanoString Technologies to design probes targeting all transcripts of each of the genes of interest. The RNA samples were diluted in RNase-free water to give a total RNA concentration of 40 ng/μL. The 12-assay nCounter master kit (NanoString Technologies, 100052, Seattle, WA, USA) was used and the experiment performed on the nCounter Analysis system (NanoString Technologies, NCT-SYS-120, Seattle, WA, USA) according to the manufacturer’s instructions at the University of Auckland Grafton Clinical Genomics facility. The nSolver Analysis software (version 4.0; NanoString Technologies) was used to adjust the raw gene expression data for background noise by thresholding to negative controls, and to normalize the data based on positive controls and the housekeeping genes. The normalized gene expression data was then graphed using Prism (version 7; GraphPad Software).

## 5. Conclusions

The α5GABAA receptors are preferentially expressed in the hippocampus where they mediate tonic inhibition and are thus thought to be crucial for memory and cognitive processes. Inverse agonists of α5GABAA receptors have been shown to improve hippocampal-dependent learning and memory with minimal off-target effects. Our study is the first to show that α5IA, an inverse agonist of α5GABAA receptors, is able to prevent Aβ_1–42_-induced cell loss in vitro, indicating that it may have neuroprotective properties. Additionally, we have shown that Aβ induces subtle changes in the expression of genes encoding GABAAR subunits. Importantly, we have shown that targeting α5GABAARs with α5IA reverses the Aβ-induced α5 subunit upregulation and reduces cell death, a key pathological feature of AD. Targeting these receptors might enable us to ameliorate the imbalance in excitatory/inhibitory neurotransmission, prevent cell death, and potentially have a disease-modifying effect in AD. Thus, α5GABAAR inverse agonists might represent a novel avenue in the development of clinical strategies to combat neuronal cell death, which could lead to improved functional outcomes in AD patients, including enhanced memory and cognition.

## Figures and Tables

**Table 1:** Summary of changes in expression of GABAergic signaling components in mouse primary hippocampal cultures following treatment with 1 nM Aβ_1–42_ and/or 100 nM α5IA.

Component	1 nM Aβ_1-42_ vs. No Treatment Control	100 nM α5IA + 1 nM Aβ_1-42_ vs. 1 nM Aβ_1-42_
**GABAA receptor subunits**		
GABAAR α1	−	−
GABAAR α2	↑	−
GABAAR α3	−	−
GABAAR α4	−	−
GABAAR α5	↑↑	↓
GABAAR α6	−	−
GABAAR β1	−	−
GABAAR β2	↑	−
GABAAR β3	↑	−
GABAAR δ	−	−
GABAAR ε	−	−
GABAAR γ1	−	−
GABAAR γ2	−	−
GABAAR γ3	−	−
GABAAR π	−	−
GABAAR θ	−	−
**GABAB receptor subunits**		
GABABR 1	↑	−
GABABR 2	↑	−
**GABA transporters**		
BGT1	−	−
GAT 1	−	−
GAT2	−	−
GAT3	−	−
VGAT	−	−
**GABA synthesizing and catabolizing enzymes**		
GAD65	−	−
GAD67	−	−
Embryonic GAD	−	−
ABAT	−	−

↑ increase, ↓ decrease. Number of arrows indicates level of significance.

**Table 2:** List of genes encoding various components of the GABAergic system selected for RNA expression analysis using the NanoString nCounter system. Housekeeping genes are indicated with an asterisk.

Gene Symbol	Gene Name	Accession Number
AARS*	Alanyl-tRNA synthetase	NM_146217.4
ABAT	GABA transaminase	NM_001170978.1
ACTB*	Beta-actin	NM_007393.3
ASB7*	Ankyrin repeat and SOCS box containing 7	NM_080443.2
CCDC127*	Coiled-coil domain containing 127	NM_024201.3
CNOT10*	CCR4-NOT transcription complex subunit10	NM_153585.5
GABBR1	GABAB receptor 1 subunit	NM_019439.3
GABBR2	GABAB receptor 2 subunit	NM_001081141.1
GABRA1	GABAA receptor α1 subunit	NM_010250.4
GABRA2	GABAA receptor α2 subunit	NM_008066.3
GABRA3	GABAA receptor α3 subunit	NM_008067.4
GABRA4	GABAA receptor α4 subunit	NM_010251.2
GABRA5	GABAA receptor α5 subunit	NM_176942.4
GABRA6	GABAA receptor α6 subunit	NM_001099641.1
GABRB1	GABAA receptor β1 subunit	NM_008069.4
GABRB2	GABAA receptor β2 subunit	NM_008070.3
GABRB3	GABAA receptor β3 subunit	NM_008071.3
GABRD	GABAA receptor δ subunit	NM_008072.2
GABRE	GABAA receptor ε subunit	NM_017369.2
GABRG1	GABAA receptor γ1 subunit	NM_010252.4
GABRG2	GABAA receptor γ2 subunit	NM_177408.5
GABRG3	GABAA receptor γ3 subunit	NM_008074.2
GABRP	GABAA receptor π subunit	NM_146017.3
GABRQ	GABAA receptor θ subunit	NM_020488.1
GAD1_1	Glutamic acid decarboxylase (GAD)67	NM_008077.5
GAD1_2	Embryonic GAD	NM_001312900.1
GAD2	GAD65	NM_008078.2
GAPDH*	Glyceraldehyde-3-phosphate dehydrogenase	NM_008084.2
SLC32A1	Vesicular GABA transporter (VGAT)	NM_009508.2
SLC6A1	GABA transporter (GAT) 1	NM_178703.4
SLC6A11	GAT3	NM_172890.3
SLC6A12	Betaine transporter 1 (BGT1)	NM_133661.3
SLC6A13	GAT2	NM_144512.2

*Statistical analysis.* Data in all experiments were expressed as mean ± SEM. One-way ANOVA followed by Bonferroni’s post hoc test was used to examine the differences between multiple groups for all experiments. All statistical analyses were conducted using Prism (version 7; GraphPad Software) with a value of p ≤ 0.05 accepted as statistically significant.

## Abbreviations

α5GABAARs	α5-subunit containing GABAA receptors
ABAT	4-aminobutyrate aminotransferase
Aβ1–42	Amyloid beta
AD	Alzheimer’s disease
ANOVA	Analysis of variance
APP	Amyloid precursor protein
araC	Cytosine arabinofuranoside
BGT-1	Betaine transporter
CC3	Cleaved caspase 3
DIV	Days in vitro
DMCM	methyl 6,7-dimethoxy-4-ethyl-β-carboline-3-carboxylate
E/I	Excitatory/Inhibitory
ELISA	Enzyme linked immunosorbent assay
FDA	Food and drug administration
GABA	γ-aminobutyric acid
GABAAR	GABAA receptors
GABABR	GABAB receptors
GABA-T	GABA transaminase
GAD	Glutamic acid decarboxylase
GAT-1	GABA transporter 1
GAT-2	GABA transporter 2
GAT-3	GABA transporter 3
PDL	Poly-D-lysine
P0	Postnatal day 0
RT	Room temperature
SEM	Standard error of mean
VGAT	Vesicular GABA transporter
